# Effect of TiC Particles on High-Temperature Properties of Al-Li Alloy and Coarsening of Al_2_CuLi(T_1_) Precipitates

**DOI:** 10.3390/ma18050922

**Published:** 2025-02-20

**Authors:** Zaihong Wang, Zhuoyang Ren, Yong Li, Zhao Wang, Jialin Chen, Zhexu Sun, Zhihao Wang, Haiyao Wang, Hongqun Tang

**Affiliations:** 1 School of Materials Science and Engineering, Northeastern University, Shenyang 110819, China; 18742418590@163.com (Z.W.); 17342343076@163.com (Z.R.); 13002408263@163.com (Z.W.); jialin10112004@163.com (J.C.); szxcnln@163.com (Z.S.); 20233912@stu.neu.edu.cn (Z.W.); 2 State Key Laboratory of Rolling and Automation, Northeastern University, Shenyang 110819, China; wanghyneu@126.com; 3 Guangxi Advanced Aluminium Processing Innovation Center Co., Ltd., Nanning 530007, China; 4 State Key Laboratory of Featured Metal Materials and Life-Cycle Safety for Composite Structures, Nanning 530004, China; hqtang@gxu.edu.cn

**Keywords:** aluminium–lithium alloy, TiC particles, coarsening of T_1_ phase, high-temperature performance

## Abstract

This study investigates the effect of TiC particles regarding the properties of aluminium–lithium alloys under high-temperature conditions, focusing on the reinforcing effect of TiC and TiB_2_ particles in the aluminium matrix and the effect on the coarsening process of T_1_ precipitates. Aluminium–lithium alloys are widely used in aerospace applications, especially as skin materials for fast vehicles, due to their excellent high specific strength and corrosion resistance. However, conventional aluminium alloys are inadequate in meeting the elevated temperature service requirements associated with supersonic flight. Consequently, there is a significant scientific imperative to investigate the heat resistance of novel aluminium–lithium alloys. The inclusion of TiC and TiB_2_ nanoparticles has been demonstrated to enhance the mechanical properties of the alloys, particularly at high temperatures of 200 °C. These particles have been shown to enhance the strength and toughness of the alloy through mechanisms such as grain refinement and increased dislocation density. Concurrently, this study determined that the coarsening phenomenon of T_1_ precipitates occurs at elevated temperatures. The inclusion of TiC particles, however, has been shown to inhibit the coarsening process, delay the coarsening of the T_1_ phase, and enhance the mechanical properties of the material. This outcome is of considerable significance for the composition design of aluminium–lithium alloys and their performance optimisation in high-temperature applications.

## 1. Introduction

Aluminium–lithium alloys are one of the key materials that are indispensable for today’s spacecraft and aircraft fuselages. It has been reported that with the addition of every 1 per cent of lithium, the density of the alloy drops by approximately 3 per cent while the modulus rises by around 6 per cent [1]. Aluminium–lithium alloys are extensively utilised in the fabrication of airframe forgings, particularly in the context of aircraft skins, because of their extraordinary properties of high specific strength, high specific stiffness, high toughness, excellent corrosion resistance, and prolonged service life. Supersonic flight, defined as travel at speeds in excess of Mach 1.2, represents a highly advanced domain within the realm of modern aeronautical technology [2]. Aluminium–lithium alloys are extensively utilised in the fabrication of airframe forgings, particularly in the context of aircraft skins, on account of their extraordinary characteristics, including high specific strength, high specific stiffness, high toughness, superb corrosion resistance, and prolonged service life. Supersonic flight, defined as travel at speeds in excess of Mach 1.2, represents a highly advanced domain within the realm of modern aeronautical technology [2,3]. Consequently, the fabrication of aluminium–lithium alloys with elevated heat-resistant temperatures through the design of components and the examination of their mechanical properties under high-temperature service conditions is of paramount importance for the augmentation of heat-resistant performance and the safety of aircraft skins for supersonic vehicles [3].

Considerable attention has been attracted by the development of particle-reinforced aluminium matrix composites in the automotive and aerospace sectors. The reasons for this are twofold. Firstly, the composites exhibit excellent properties, such as low density, high strength, good creep and wear resistance, and higher stiffness compared to conventional aluminium alloys [3]. Among these composites, those with an aluminium alloy matrix and silicon carbide (SiC) as reinforcing particles have emerged as leaders. Although continuous fibre-reinforced metal matrix composites (MMCs) exhibit excellent mechanical properties [4], nevertheless, particle-reinforced MMCs have superior processability. The majority of research in this domain has concentrated on micro-sized particle enhancement. However, the potential of nano-sized SiC particles to achieve high strength with reasonable toughness in an aluminium matrix with a homogeneous microstructure is a current area of active exploration [5].

In accordance with the nature of the reinforcement, aluminium matrix composites can be categorised into four distinct classifications, namely (a) particle-reinforced aluminium matrix composites [6]; (b) whisker- or staple fibre-reinforced aluminium matrix composites; (c) continuous fibre-reinforced aluminium matrix composites; and (d) monofilament fibre-reinforced aluminium matrix composites. This paper focuses on the use and presentation of particle-reinforced aluminium matrix composites [7].

Particle-reinforced aluminium matrix composites are prepared at low cost. Although the mechanical properties of particle-reinforced aluminium matrix composites may not be comparable to those of aluminium matrix composites reinforced by other methods [8], however, its properties are far superior to those of unreinforced aluminium alloys [9]. Furthermore, particle-reinforced aluminium matrix composites are isotropic and thus possess the capacity to withstand a variety of secondary forming operations, including extrusion, rolling, and forging [10].

Key parameters for the development of these composites include the volume fraction and size of reinforcing particles [11]. Increasing the particle volume fraction increases the strength but may decrease the toughness due to localised deformation [12,13]. Conversely, reducing particle size increases both strength and toughness, as smaller particles are less likely to fracture. In addition, particles can pinch grain boundaries, stabilise substructural units, accelerate the ageing process, and increase the work hardening rate [14]. Typically, particle-reinforced aluminium matrix composites employ isometric ceramic particles with an aspect ratio of less than 5 as a form of reinforcement. These ceramic reinforcement phases are typically oxides, carbides, nitrides, or borides [15], such as Al_2_O_3_, SiC, TiC, Al_3_Ti, Al_3_Zr, AlN, or TiB_2_.

TiC has proven to be an excellent ceramic particle [16], with high hardness and wear resistance and the same face-centred cubic structure as the Al matrix, resulting in excellent crystal structure compatibility [17]. Unlike TiB_2_, TiC particles are smaller in size, usually between 50 nm and 100 nm. This smaller particle size allows composites containing TiC particles to exhibit relatively better toughness while maintaining high strength [18,19]. This means that these composites are not only tough and wear-resistant but also better absorb energy when subjected to external forces, reducing the risk of brittle fracture and thus improving the overall structural integrity and longevity.

Conventional aluminium–lithium alloys are designed for high strength by increasing the alloying element content and complex microalloying. Al-Cu-Li alloys are widely used [20] because their complex microalloying facilitates the deposition of the T_1_ (Al_2_CuLi), δ′ (Al_3_Li), and θ′ (Al_2_Cu) phases, which improves the strength of Al-Li alloys [21]. In general, the main strengthening effect in Al-Cu-Li alloys comes from the T_1_ (Al_2_CuLi) phase that precipitates along the {111} facets of the Al matrix and tends to nucleate at grain or subgrain boundaries [12]. In their study, Shao et al. [22] observed that the incorporation of in situ TiB_2_ particles and the addition of Ti solutes significantly enhanced the physical properties of the composites, including the modulus of elasticity and hardness, while concomitantly accelerating the ageing process, thereby reducing the time required to attain peak properties. While the TiB_2_ particles exhibited a limited impact on the δ′ precipitates, they were found to promote the nucleation of the T_1_-Al_2_CuLi precipitates and enhance the homogeneity and fineness of the precipitates. Concurrently, TiB_2_ particles promoted the growth of δ′-PFZ (precipitation-free zone), while Ti solutes significantly inhibited its broadening, and the reduced ageing temperature enhanced this inhibition. Consequently, the collective impact of these additives resulted in a substantial enhancement of the mechanical properties of the composites. This finding provides a foundation for performance optimisation through a comprehensive analysis of the underlying mechanisms.

A variety of precipitates, including T_1_, θ′, S and σ phases, have been observed in Al-Li alloys following ageing treatment. Among these precipitates, T_1_ (Al_2_CuLi) exhibits a lamellar morphology and a hexagonal structure, which is regarded as the main strengthening phase in Al-Cu-Li alloys, along with being the predominant source of alloy strength. Cui et al. [23] examined the precipitation behaviour of Al-Cu-Li-X alloys under various ageing conditions. The process of T_1_ precipitation is challenging and relies heavily on the involvement of delocalisation. Deng et al. [17] investigated the formation mechanism of T_1_ precipitates and demonstrated that the formation of T_1_ precipitates requires the involvement of delocalisation. Li et al. [24] showed that the addition of nanoscale TiC and TiB_2_ particles to Al-Cu-Li alloys can effectively promote the precipitation of the T_1_ phase, thereby improving the mechanical properties of the alloys. It has been demonstrated that the mechanical properties of the alloy are improved by this process. Furthermore, it has been demonstrated that performing plastic deformation before ageing serves as an effective method to enhance the nucleation and growth of T_1_ precipitates. In addition, Wu et al. [25] investigated the precipitation process of TiB_2_/Al-Li-Cu composites and found that TiB_2_ ceramic particles promoted the phase precipitation of θ′ and T_1_. Finally, Agustianingrum et al. [26], utilising Thermo-Calc software, performed thermodynamic calculations, which predicted that the T_1_ phase is stable up to 427 °C in the AA2195 alloy, while in the Al-2.5Cu-2Li and Al-3Cu-1.5Li alloys, the T_1_ phase is stable in the temperature range of 467–517 °C and 377–507 °C, respectively. However, according to experimental observations, at an ageing temperature of 170 °C, the alloy T_1_, θ′, and δ′ phases exist, which are below the predicted stabilisation range of the T_1_ phase. It has been established that the T_1_ phase has the capacity to precipitate at temperatures that fall below the anticipated stability range. Furthermore, it has been demonstrated that the T_1_ phase exhibits a decline in stability in comparison to other phases, such as θ′(Al_2_Cu) and S(Al_2_CuMg), at temperatures reaching approximately 200 °C. This decline is most likely attributable to the high-temperature coarsening of the T_1_ precipitates. Wu et al. [27] observed that the T_1_ precipitates undergo substantial coarsening at temperatures up to 175 °C. At temperatures above 175 °C, the T_1_ precipitates began to coarsen significantly, with approximately a 30% increase in the diameter and a substantial decrease in number density. The underlying cause for this coarsening is believed to be the thickening of the T_1_ precipitates, a process facilitated by the enhanced diffusivity of the Cu atoms at higher temperatures, as shown by Z. Gao et al. [28]. It has been demonstrated that the T_1_ precipitates nucleate directly from their own GP (Guinier Preston) region and grow thicker in two possible ways: by repeating the T_1_ unit cell in the conventional way or by the anomalous way of T_1_ variants. Furthermore, Li et al. [24] found that the growth of T_1_ precipitates occurs on the <111>_Al_ crystal plane with two different growth orientations: one along the length direction and the other along the thickness direction. In a related study, Kang et al. utilised corrected scanning transmission electron microscopy (STEM) and energy-dispersive X-ray spectroscopy (EDX) to investigate the atomic structure of T_1_ precipitates in Al-Li-Cu-Mg alloys and the growth mechanism [29]. Their study revealed that T_1_ precipitates can be thickened and thus grown by successive stacking of identical 0.94 nm thick layers.

In this study, Al-Li alloys containing TiC and TiB_2_ particles were prepared by stir casting and the high-temperature tensile properties of the alloys at 200 °C were investigated. Furthermore, the coarsening process of T_1_ precipitate growth under high-temperature thermal exposure and the effect of TiC and TiB_2_ particles on the coarsening process were also studied. The findings of this study can provide a reference for the composition design and performance study of aluminium–lithium alloys under high-temperature service.

In particular, the experimental methodology in this study builds upon the foundational work of Wang et al. [4] and Li et al. [24], who systematically investigated the role of nano-TiC/TiB2 particles in promoting T_1_ precipitation and optimising ageing kinetics in Al-Cu-Li alloys. While their research focused on room-temperature precipitation behaviour and mechanical property enhancement, the present work extends these principles to high-temperature service conditions (200 °C), specifically examining T_1_ coarsening mechanisms and their correlation with tensile performance under thermal exposure. This methodological continuity ensures scientific rigour while addressing a critical gap in high-temperature stability analysis.

## 2. Experimental Section

### 2.1. Material and Sample Preparation

Table 1 presents the alloy compositions of the three designed and actual alloys, which are denoted as 2055, 0.5, and 1. The raw materials included pure aluminium with a purity of 99.7%, pure zinc with 99.9% purity, pure magnesium at 99.9% purity, pure lithium with 99.8% purity, pure silver with 99.9% purity, and the intermediate alloys [30] (Al-50Cu, Al-5Zr, Al-10Mn, and Al-30TiC/TiB_2_). The fractions of TiC/TiB2 particles were selected as 0.5% and 1.0% [31], based on the following considerations: (1) Previous molecular dynamics simulations show that when the TiC volume fraction is 0.3–0.6%, the dislocation-particle interaction energy is maximised [30]. (2) The seepage theory predicts that the seepage threshold of TiC in the aluminium matrix is about 1.2 vol%, and the amount of addition (0.38–0.59 vol%) in this experiment is lower than that of this threshold is to avoid the risk of brittleness caused by particle agglomeration. (3) The optimisation of the stirred casting process can inhibit local agglomeration of 1.0% samples, ensuring that the strengthening effect is dominated by the dominant force. The preparation of the TiC/TiB_2_ intermediate alloy was carried out in accordance with the references [9], where the melt was maintained at 720–750 °C for 3 h. The TiC/TiB_2_ intermediate alloy was maintained at 300 °C for 1–2 h. The preheated TiC/TiB_2_ intermediate alloy was added to the alloy at 730 °C and stirred for 15 min at 80 rpm by using a machine, and C_2_Cl_6_ was used as the degassing agent. The degassing temperature was 720 °C and the pouring temperature was 710 °C. Then, the molten mixture was poured into a water-cooled copper mould, where the size of the copper ingot was 200 × 150 × 30 mm [32]. Table 2 lists the various properties of the two main types of reinforcement particles, TiC and TiB_2_.

### 2.2. Heat Treatment and Rolling Process

To eliminate the residual stresses and homogenise the cast structure, the cast ingots were first homogenised at 490 °C for 24 h. Subsequently, they were hot-rolled to a thickness of 3 mm (from 30 mm to 3 mm). The rolled plates were solution-treated at 500 °C for 15 min and then quenched with cold water. Immediately after that, the plate samples were heat-treated at 200 °C for 24 h and 48 h.

### 2.3. Microstructure Characterisation and Property Tests

To analyse the microstructure of the ingots, a JEOL JXA—8530F (JEOL, Akishima, Japan) on-site electron probe microanalysis (EPMA) was utilised. A ZEISS ULTRA 55 (ZEISS, Oberkochen, Germany) field emission scanning electron microscope (SEM) with electron backscatter diffraction (EBSD) attachment was used. The EBSD samples were prepared via electrochemical polishing. They were polished at 25 V for 30 s in an electrolyte, which was a mixture of 10% HClO_4_ and 90% C_2_H_5_OH.

Transmission electron microscope (TEM) analysis was carried out using a TECNAI G2 F20 system (FEI Corporation of America, Columbia, MD, USA) operating at 200 kV. The samples for TEM were double-spray electropolished in a solution composed of 30% HNO_3_ and 70% CH_3_OH at 25 °C with a polishing voltage of 15 V. The stretching performance was evaluated at room temperature. An Instron—4206 100 kN universal testing machine was employed at a rate of 2 mm/min. To ensure data accuracy, three parallel samples were prepared for each tensile testing group [33].

## 3. Results

**Figure 1:**
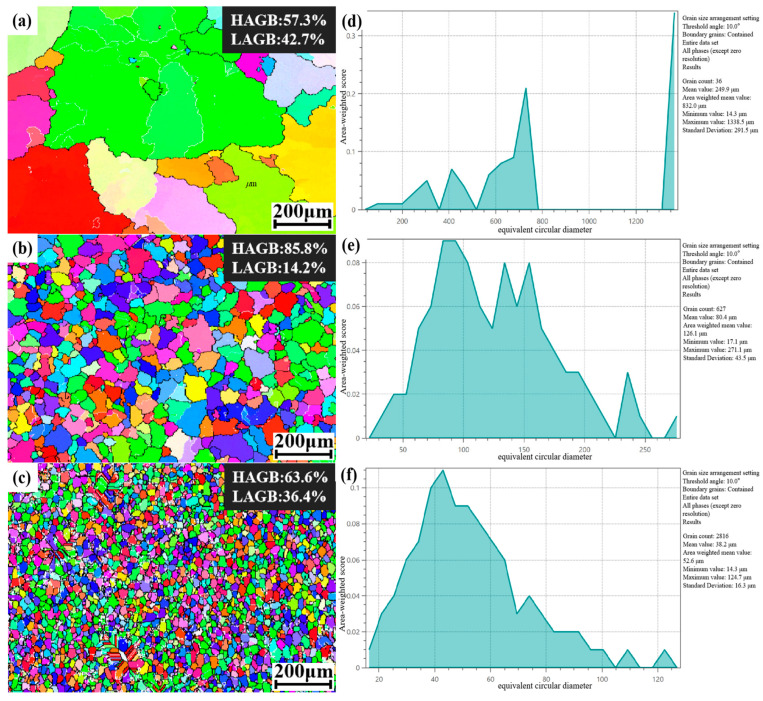
Cast EBSD results for samples: (**a**) A_0_; (**b**) A_0.5_; (**c**) A_1_. Equivalent circle diameter statistical results: (**d**) A_0_; (**e**) A_0.5_; (**f**) A_1_.

Figure 1a,b,c illustrate the IPF maps of the as-cast A_0_, A_0.5_, and A_1_ alloys obtained by EBSD. It is evident that the grain sizes of these three alloys differ significantly. The grain size distribution of the A_0_ alloy ranges from 200 to 1400 μm, with an average grain size of 249. 89 μm. The grain size distribution of the A_0.5_ alloy ranges from 40 to 250 μm, with an average grain size of 86.42 μm. The grain size distribution of the A_1_ alloy ranges from 14 to 124 μm, with an average grain size of 38.2 μm. Additional TiC + TiB_2_ particles have been shown to significantly refine the grain size, with the degree of grain refinement increasing in proportion to the number of added TiC + TiB_2_ particles. Furthermore, the degree of grain refinement of the A_1_ sample with 1.0% TiC + TiB_2_ is significantly higher than that of the A_0.5_ sample with 0.5% TiC + TiB_2_ [34].

**Figure 2:**
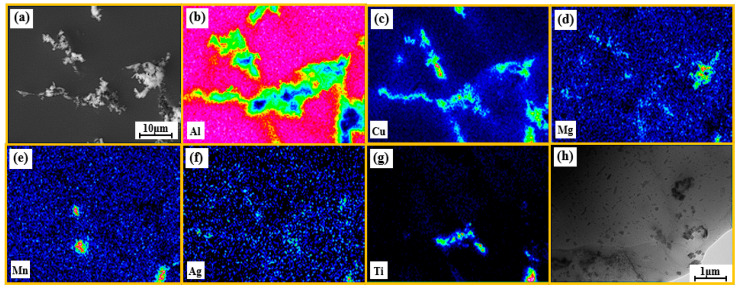
(**a**–**g**) Cast SEM micrographs and EDS elemental mapping analysis of A_1_ samples; (**h**) TEM images of TiC and TiB_2_ particles in A_1_ alloy.

The electron probe microanalysis (EPMA) technique was utilised to ascertain the distribution of TiC and TiB_2_ particles in the as-cast A_1_ sample, in addition to the spatial distribution state of various other elements. The results are presented in Figure 2a–g. The microstructure of the alloy consists of α-Al (α-Aluminium Oxide) grains and their intergranular second phase, which results from the non-equilibrium solidification produced during the casting process (Figure 2a). Notably, dark flocculated phases were observed under EPMA, and the presence of elemental Ti in these phases was further confirmed by subsequent energy-dispersive X-ray spectroscopy (EDS) analysis (Figure 2g), which suggests that the observed dark phases are TiC and TiB_2_ particles aggregated at the α-Al grain boundaries. In addition, the EDS results indicate that the Al element is hardly enriched at the grain boundaries (Figure 2b), and the other three main alloying elements (Cu, Mg, and Mn) undergo a more pronounced enrichment at the grain boundaries and in the flocculated second phase (Figure 2c–e). Conversely, the Ag element exhibits a more uniform distribution in the as-cast A_1_ sample. The sizes of TiC and TiB_2_ particles in the as-cast A_1_ samples were observed to be approximately 20–120 nm under transmission electron microscopy (TEM) (Figure 2h).

**Figure 3:**
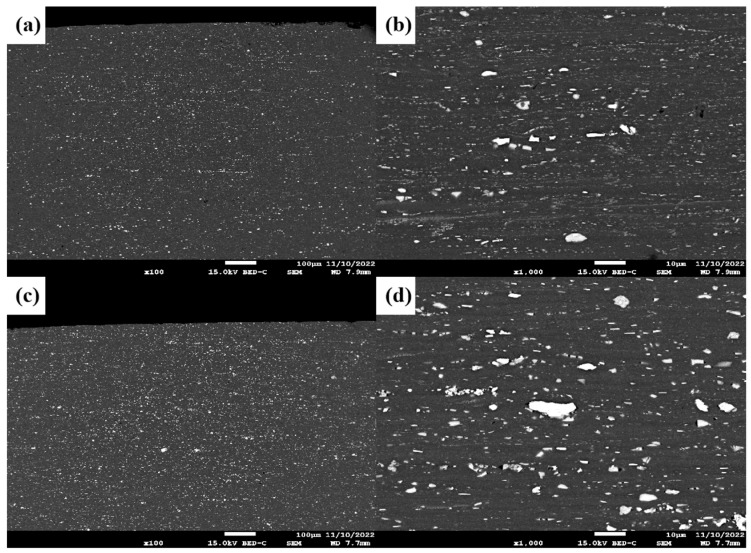
SEM micrographs of hot-rolled state: (**a**) A_0_ specimens 100 μm; (**b**) A_0_ specimens 10 μm; (**c**) A_1_ specimens 100 μm; (**d**) A_1_ specimens 10 μm.

Figure 3 illustrates the metallographic organisation and the distribution of eutectic phase particles in the hot-rolled state. It is evident from the figure that after the hot-rolling treatment, the metallographic organisation of the alloy underwent significant changes. The thermal deformation and dynamic recrystallisation during the hot-rolling process resulted in the large grains present in the as-cast state being broken down more finely, thereby forming a uniform and fine grain structure [35]. As demonstrated in Figure 3a, the grains of the A_0_ alloy have undergone substantial refinement following hot rolling in comparison with their organisation in the cast state. Figure 3b illustrates that subsequent to hot rolling, the internal grains of the alloy are distinctly distributed along the streamlined direction of rolling, thereby forming a transverse weaving structure. The precipitated T_1_ phases are distributed around the grain boundaries with a more uniform distribution. Figure 3c illustrates that the TiC and TiB_2_ particles in the hot-rolled A_1_ sample are predominantly aggregated at the α-Al grain boundaries, and their distribution is more diffuse and homogeneous compared with that of the as-cast alloy. With the addition of 1.0% TiC + TiB_2_ nanoceramic particles, the number of T_1_ phases precipitated between grain boundaries is increased, as illustrated in Figure 3d. Figure 3d demonstrates that the T_1_ phases between grain boundaries are more substantial in the alloy with the addition of nanoceramic particles in comparison to the A_0_ sample. This suggests that the incorporation of nanoceramic particles can effectively promote the precipitation and growth of the T_1_ phase in the hot-rolled state [36].

**Figure 4:**
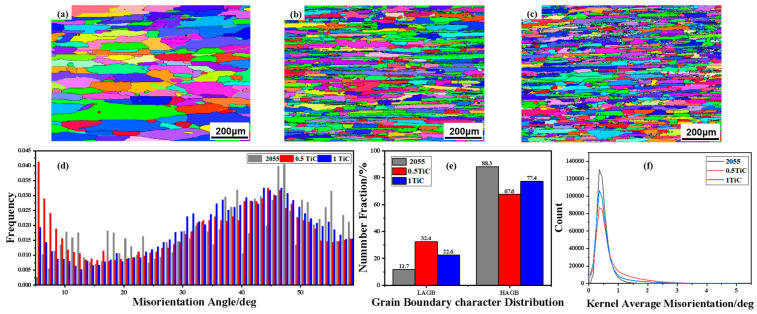
EBSD results of alloys after solution treatment: (**a**) A_0_; (**b**) A_0.5_; (**c**) A_1_; (**d**) misorientation angle distribution; (**e**) grain boundary character distribution; (**f**) kernel average misorientation.

The results of the EBSD analysis of the A_0_, A_0.5_, and A_1_ alloys subjected to solid solution treatment after rolling deformation are presented in Figure 4. Following the complete recrystallisation treatment at 500 °C, these alloy bands exhibit significant microstructural evolution. It is noteworthy that the recrystallised grain sizes of the A_0.5_ and A_1_ alloys are significantly smaller than those of the A_0_ alloy (Figure 4a,b). This phenomenon can be attributed to the smaller as-cast grain size, which contributes to the formation of more grain boundaries in the matrix, thus providing more nucleation sites for the recrystallisation process. Furthermore, Figure 4b,c shows the recrystallised grains of A_0.5_. The arrangement of the recrystallised grains of the A_0.5_ and A_1_ alloys is in parallel along the rolling direction (Figure 4b,c). Although a similar phenomenon is observed for the A_0_ alloy (Figure 4a), this alignment orientation is less pronounced for the A_0.5_ and A_1_ alloys due to their larger grain size. It has been shown that the particle-stimulated nucleation (PSN) effect during the recrystallisation of aluminium alloys is promoted by TiC and TiB_2_ ceramic particles [37]. During the process of rolling deformation, the eutectic phases that are present at grain boundaries following homogenisation are disrupted and distributed in chains along the rolling direction. The inducibility of the PSN effect is contingent upon the critical nucleation size, and the relationship can be described by the equations that have been previously described in the literature.

In the presence of particles, the net driving force for recrystallisation can be expressed as

(1) $$P=P_{D}-P_{Z}=\frac{\alpha \rho Gb^{2}}{2}-\frac{3F_{v}\gamma_{b}}{d_{p}}$$

In the formula, $\gamma_{b}$ is the specific grain boundary energy of aluminium alloys, and its value is 0.32 J/m^2^; $P_{D}$ and $P_{z}$ denote, respectively, the deformation storage energy exerted by the fine particle and Zenner pinning force; α is a constant, which is valued at 0.5; G is the modulus of rigidity, which is valued at 25.5 GPa; b indicate Burgers vectors.

The critical size required for nucleus growth is defined as

(2) dcrit=4γbPD−PZ=4γbαρGb22−3Fvγbdp

As illustrated in Figure 4d, the statistical outcomes concerning the orientation deviation angles in the A_0_, A_0.5_, and A_1_ alloy matrices are presented. It is evident from Figure 4d that the A_0.5_ alloy exhibits a greater prevalence of orientation deviation angles less than 10° (i.e., small-angle grain boundaries) subsequent to solution treatment at 500 °C, signifying that the A_0.5_ alloy contains a greater number of small-angle grain boundaries, and it is well established that these represent a greater number of dislocations. Therefore, the dislocation density in the A_0.5_ alloy is very high, and there is a larger percentage of large-angle orientation deviation angles in the A_1_, indicating that the A_1_ alloy has more large-angle grain boundaries, and the dislocation density is clearly smaller than that in the A_0_. The distribution of grain boundary features in the A_0_, A_0.5_, and A_1_ alloys is illustrated in Figure 4e, with the A_0.5_ alloy exhibiting a reduced number of large-angle grain boundaries and an increased number of small-angle grain boundaries in comparison to the A_1_ and A_0_ alloys. Figure 4f reflects the average angle of dislocations less than 5° in the three alloys. This is attributable to the post-rolling grain growth by the recrystallisation process during solid solution treatment after rolling [38].

**Figure 5:**
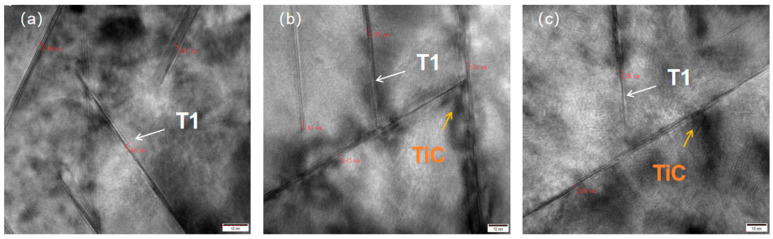
DM analysis of T_1_ phase and ceramic particle size thickness: (**a**) A_0_; (**b**) A_0.5_; (**c**) A_1_.

Size/thickness analysis of the T_1_ phase and ceramic particles using TEM at high resolution in Figure 5 showed that in the over-aged sample A_0_ without ceramic particles in Figure 5a, the size of the T_1_ phase varied from 2.6 to 3.0 nm, with an average size of about 2.856 nm; the average sizes of the over-aged A_0.5_ in Figure 5b and A_1_ in Figure 5c with ceramic particles were about 2.66 nm and 2.47 nm, respectively, with the size of the TiC ceramic particles varying from 4 to 11 nm. The ceramic particles have an obvious inhibiting effect on the coarsening of the T_1_ phase, and the inhibiting effect becomes more obvious with the increase in the ceramic particles.

**Figure 6:**
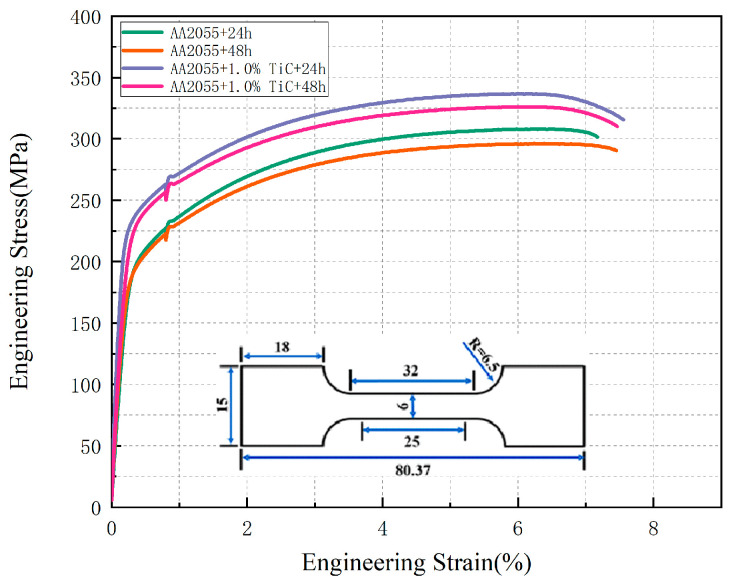
High-temperature tensile stress–strain curve.

A series of high-temperature tensile experiments were conducted on four samples at 200 °C, and the resultant stress–strain curves are depicted in Figure 6. The samples were subjected to an ageing treatment at 200 °C for 24 h, resulting in a yield strength of 223 MPa and a tensile strength of 308 MPa. An extension of the ageing treatment to 48 h led to a yield strength of 217.5 MPa and a tensile strength of 296 MPa. The addition of 1% TiC to the ageing treatment at 200 °C for 24 h resulted in a yield strength of 257 MPa and a tensile strength of 336 MPa. Finally, the incorporation of 1% TiC to the ageing treatment at 200 °C for 48 h yielded a yield strength of 250.5 MPa and a tensile strength of 326 MPa.

It has been demonstrated that the yield and tensile strengths of samples of the same composition decrease under excessively long ageing treatments. This phenomenon may be attributed to the significant evolution of the microstructure of AA2055 due to prolonged thermal exposure. Typical T_1_ (Al_2_CuLi) precipitates are significantly coarsened, while their number density decreases significantly [39]. Concurrently, the T_1_ precipitates were gradually replaced by coarser δ′ and S phases with increasing temperature. The coarsening of the precipitates reduces the hindrance to dislocation motion, which leads to a decrease in strength and toughness.

For the samples with different compositions, as shown in Table 3, the addition of TiC particles increased the yield and tensile strengths of the samples: the yield strength increased by 15.2% and tensile strength increased by 9% compared to the specimens without ceramic particles under ageing conditions at 24 h and 200 °C. Also, the yield strength increased by 15.1% and tensile strength increased by 10% compared to the specimens without TiC particles under ageing conditions at 48 h and 200 °C. This proves that the addition of TiC particles has a positive effect on the strength and toughness of the alloy, and this positive effect does not change with long-term heat exposure and has strong stability. This enhancement in strength and toughness may be attributed to the fact that in alloys containing TiC particles, the coarsening of T_1_ precipitates is inhibited due to the presence of ceramic particles. The TiC particles act as barriers to the migration and aggregation of the T_1_ precipitates, leading to a finer and more stable microstructure. TiC particles likewise increase the dislocation density in the alloys, thus contributing to dislocation strengthening. In addition, these TiC particles promote the emergence of Orowan dislocation rings, which further increase the yield strength, and TiC particles distributed at grain boundaries inhibit crack propagation and improve toughness [40].

**Figure 7:**
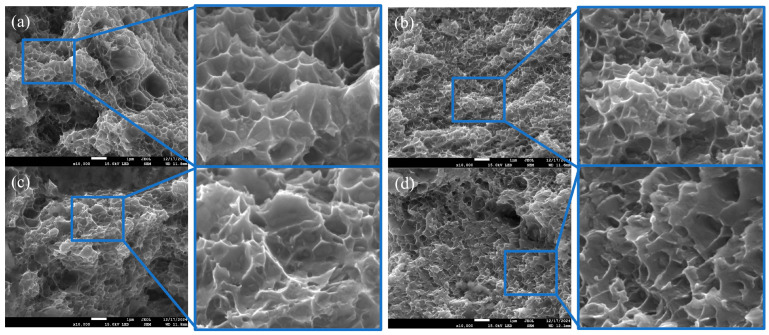
SEM fracture topography of four samples: (**a**) A_0_ + 24 h, (**b**) A_1_ +24 h, (**c**) A_0_ + 48 h, (**d**) A_1_ +48 h.

The fracture surface of the specimen post-tensile experiment was analysed using SEM, as illustrated in Figure 7. During the fracture process of the specimen and when subjected to external forces, microscopic plastic deformation occurs. In this process, micropores inside the specimen nucleate at the location of the second-phase particles, inclusions, etc. These micropores then continue to grow and gather, ultimately leading to the fracture of the specimen. As depicted in Figure 7a, the tough nest exhibits a substantial size and irregular shape, with prominent second-phase particles present at its base. The depth of the tough nest also demonstrates a significant variation. In Figure 7b, the ligamentous fossa manifests a reduced size and more regular shape, with the second-phase particles at the base being smaller and more dispersed [41]. It is hypothesised that this phenomenon is attributable to the presence of TiC grains as a heterogeneous phase within the aluminium alloy matrix. This presence provides a greater number of locations for the nucleation of ligamentous nests, thereby increasing the number of ligamentous nests at fracture. It is further postulated that the ligamentous nests are smaller in size due to their limitation by the surrounding grains as they grow up. In this case, the ligamentous fossae on the fracture show fine and uniform characteristics. These small and dense ligament fossae can absorb more energy during the fracture process of the material. The crack primarily extends around the particles during the fracture process, thereby minimising the interference of the crack extension path. Consequently, the shape of the ligament fossa is less influenced by the shape of the particles, which are predominantly circular in nature. The crack extension process necessitates the destruction of these small ligament fossae individually, which consumes a specific amount of energy. This, in turn, results in a longer crack extension path and enhances the material’s toughness. The ligament fossa in Figure 7c is observed to be both larger in size and more irregularly shaped when compared to its appearance in Figure 7a. Furthermore, it has been determined that some of the second-phase particles may undergo dissolution or aggregation under the prolonged thermal exposure experienced in the experiment. If the second-phase particles are identified as the primary location for ligamentous fossa nucleation, then a decrease in their number will consequently result in a decrease in the number of ligamentous fossa nucleation sites. This, in turn, will lead to a decrease in the number of ligamentous fossae. As illustrated in Figure 7d, the ligament foci demonstrate interconnections and shallower depths, which may be attributable to the prolonged high-temperature effect that facilitates the migration of grain boundaries. The displacement of grain boundaries results in alterations to the distribution and growth direction of ligament foci. Originally isolated ligament foci may approach each other and connect due to the movement of grain boundaries. The prolonged high-temperature environment provides favourable conditions for the diffusion of vacancies and the second phase, and the diffusion of the second phase may lead to changes in the chemical composition of the local regions, making the material’s properties degrade in these regions. During the fracture process, such changes can contribute to the connection between the tough nests and the internal structural changes caused by the diffusion of vacancies and the second phase can cause the growth depth of the tough nests to be limited and then become shallower [42].

**Figure 8:**
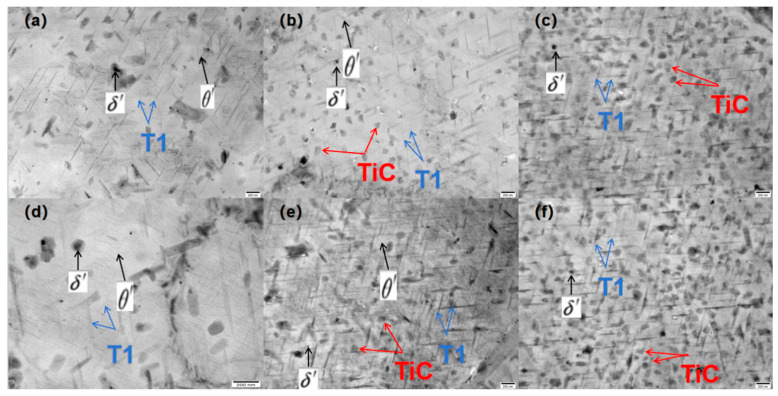
TEM fracture topography of four samples: (**a**) A_0_ + 24 h, (**b**) A_0.5_ + 24 h, (**c**) A_1_ + 24 h, (**d**) A_0_ + 48 h, (**e**) A_0.5_ + 48 h, (**f**) A_1_ + 48 h.

As illustrated in Figure 8a,d, the TEM morphology of alloy 2055 was examined after 24 and 48 h of thermal exposure, respectively. The 24 h sample exhibited a substantial number of δ′ phases, with an approximate size of 100 nm, in addition to a considerable quantity of acicular T_1_ phases, with an estimated size of around 200 nm and a modest amount of θ′ phases. Following a 24 h thermal exposure, the samples exhibited an increase in the size of the δ′ phases and T_1_ phases, indicating a short and thick morphology. Figure 8b,e illustrate the morphology of the A_0.5_ alloy; following the identical thermal exposure experiment, a specific quantity of TiC particles and a considerable number of T_1_ phases can be observed in the organisation after the 24 h treatment. Again, there is no evident change in the number of TiC particles after 24 h of thermal exposure, but the number of T_1_ phases rises considerably and is substantially higher in the sample compared to that in Figure 8a. With the changes in Figure 8d, the tendency of T_1_ phases in Figure 8e to grow to be short and thick is obviously weakened; it is no longer aggregated with each other to precipitate but instead showed the tendency to precipitate independently and separately to form more elongated T_1_ phases. The morphology and growth behaviour of the T_1_ phase of the A_1_ samples in Figure 8c,f corroborate this statement, with the introduction of more TiC particles resulting in a dramatic increase in the number of T_1_ phases in the samples. There was minimal difference in the morphology of the T_1_ after the 24 h and 48 h thermal exposures, which were elongated needle-like T_1_ phases, but with a slight increase in the number of phases. It can be hypothesised that the TiC particles will diminish the propensity of the T_1_ phases to aggregate, resulting in a greater propensity to form independent elongated needle-like T_1_ phases around the TiC particles. This is predicted to form a more complex weaving organisation and provide enhanced strength and plasticity for the samples [43].

## 4. Discussion

### 4.1. Effect of Particles on Yield Strength at High Temperatures Including Calculation of Yield Strength Contribution

In this study, we enhance the material’s high strength, low density, and excellent creep properties by introducing TiC and TiB_2_ nanoceramic particles into the AA2055 aluminium alloy matrix. According to a previous study by Li et al. [24], the microstructure of AA2055 is mainly dominated by a thin T_1_ precipitated phase. During the plastic deformation of the alloy, a significant interaction occurs between the dislocations within the AA2055 matrix and the added ceramic nanoparticles. However, the coarsening of the precipitates during ageing induces a shift from Ashby to Orowan interactions between dislocations and precipitates. Non-shearing nanoparticles effectively hinder the movement of dislocations. Upon encountering particles that are difficult for dislocations to cut through directly, the dislocations take a bypassing approach to pass through the TiC and TiB_2_ nanoceramic particles, a process that induces the formation of Orowan dislocation rings in the interfacial region between the particle population and the aluminium alloy matrix. As reported by [44], the dislocation rings that are formed increase the strain hardening capacity of the alloy. These dislocation rings exert a reverse force on the dislocation source and prevent the source from releasing dislocations. If dislocations are to be released, this resistance must be overcome, i.e., the stress must be increased, resulting in the strengthening of the material.

The obstruction of dislocations during movement is pivotal to the enhancement of strength in metallic materials, and the dislocation density is positively correlated with the strength of these materials. The relationship between intensity and dislocation density can be expressed by the Bailey–Hirsch equation:

(3) $$\Delta \sigma_{d}=M\alpha Gb\rho^{\frac{1}{2}}$$

α is a constant equal to 0.2, and ρ is the dislocation density. M is the Taylor factor. G is the shear modulus, which is valued at 25.5 GPa; b denotes the Burgers vector.

It was shown that when the precipitated phase or dispersion was bypassed by the Orowan dislocation bypass mechanism, the yield strength increment was $\Delta \sigma_{O\mathrm{rowan}}.\Delta \sigma_{O\mathrm{rowan}}$ depending on the average distance between dispersions, the relationship can be described as

(4) $$\Delta \sigma_{O\mathrm{rowan}}=\frac{0.4MGb}{\pi \sqrt{1-\nu }}\frac{\ln\frac{2\bar{\gamma }}{b}}{\lambda_{p}}=\frac{0.4MGb}{\pi \sqrt{1-\nu }}\frac{\ln\frac{2\sqrt{6}r}{3b}}{\lambda_{p}}$$

where M is the mean orientation factor with a value of 3.06; ν is the Poisson’s ratio, and its value is 0.33; $\bar{\gamma }$ is the radius of the circular cross-section of the precipitated phase in the random plane, $\bar{\gamma }=\sqrt{\frac{2}{3}}$, r is the average size of the dispersion, and $\lambda_{p}$ indicates the average distance between dispersions.

### 4.2. T_1_ Sediment Coarsening Process

During the process of high-temperature ageing, the T_1_ phase precipitates coarsen at elevated temperatures [27], thereby exerting a discernible influence on the properties of aluminium alloys at elevated temperatures. In this study, the T_1_ phase initiated the process of precipitation and growth as the ageing time was prolonged. At 24 h of ageing, the precipitation is primarily the δ′ phase of 100 nm; a substantial number of needle-like T_1_ phases with a size of approximately 200 nm and a minor amount of θ′ phase are also present. As the process progresses towards its peak, the predominant precipitate is the T_1_ phase, with a size ranging from 100 to 250 nms. This observation indicates that a sequence of reactions transpires between the T_1_ phase, the δ′ phase, and the (θ′ phase) during the transition from under-ageing to peak-ageing. In the under-ageing conditions, the δ′ phase, θ′ phase, and the T_1_ phase precipitate separately, while in the under/peak-ageing and over-ageing conditions, they grow by depleting the δ′ and θ′, which results in a sharp decrease in the number of δ′ and θ′. The roughening process of the T_1_ phase from elongated to stubby, which also occurs through the depletion of δ′ and θ′, is evident. The resulting T_1_ precipitate accumulates around the previously formed elongated T_1_ phase, causing it to grow in both width and length by roughly the same length. It is therefore clear that the width of the T_1_ phase in the aged sample is more than ten times that in the peak-aged sample. In A_0.5_ and A_1_ samples, the T_1_ precipitation formed by depleting δ′ and θ′ moves to aggregate around the original T_1_ phase and is hindered to the extent that the coarsening behaviour is weakened. The increase in the number of ceramic particles significantly enhances this hindering effect, which results in a significant reduction in the coarsening behaviour of the T_1_ phase. The presence of a significant quantity of TiC ceramic particles and a substantial number of elongated needles in the overstressed A_1_ sample is also evident. The presence of a substantial number of TiC ceramic particles and a considerable number of elongated needles in the over-aged A_1_ samples, which are occluded and hindered by each other, significantly enhances the properties of the Al-Cu-Li alloy. The introduction of ceramic particles has been shown to yield a high density of fine needle-like T_1_ phases by hindering the aggregation and coarsening of the T_1_ phases. Furthermore, the T_1_ phases themselves have been demonstrated to possess a more effective strengthening effect than the δ′ and θ′ phases, which have a positive and obvious strengthening effect on the property enhancement of Al-Cu-Li alloys [45].

In addition, combined with previous research, we can discuss the synergistic effects of TiC and TiB_2_ particles more comprehensively. We believe that it is roughly achieved through the following mechanism: (1) Grain refinement complementarity: TiB_2_, as the α-Al heteronucleation core, refines the cast grain size of A1 alloy to 38.2 μm, while TiC inhibits recrystallisation grain boundary migration through Zener pinning, stabilising the substructure. (2) T_1_ phase precipitation regulation: TEM shows that Cu atoms are enriched at the TiC/Al interface, providing a site for T_1_ phase nucleation; TiB_2_ particles induce dislocation entanglement through pre-deformation, promoting T_1_ phase edge dislocation and line precipitation, and the number density is increased by 15%. (3) High-temperature stability coordination: The thermal mismatch between the TiC (CTE = 7.4 × 10⁻⁶/K) and Al matrix (CTE = 25.5 × 10⁻⁶/K) induced interface dislocation, hindering the T_1_ phase and the solute diffusion path during roughening, while TiB_2_ suppresses the T_1_ phase by pinning the grain boundary. The above synergistic effect made 1.0% of the samples still elongate the T_1_ phase (average size 2.47 nm) after ageing at 200 °C/48 h, which was significantly better than the control group without added particles (2.85 nm).

## 5. Conclusions

1. Without TiC + TiB_2_ particles, the T_1_ phase in aluminium–lithium alloys coarsens greatly during high-temperature ageing, growing in size and decreasing in quantity. However, when TiC + TiB_2_ particles are added, they notably inhibit the T_1_ phase’s coarsening. TiC + TiB_2_ particles stop the aggregation, impeding the T_1_ phase’s movement and thus slowing its coarsening rate. The more TiC + TiB_2_ particles there are, the stronger this inhibitory effect becomes. As a result, the T_1_ phase remains finer and more uniformly distributed in the alloy, maintaining its resistance to coarsening even after long-term high-temperature exposure.
2. At 200 °C, after ageing for 24 and 48 h, an alloy with 1.0 wt.% TiC + TiB_2_ had higher yield and tensile strengths than the base alloy AA2055. For instance, the A_1_ alloy’s yield strengths were 257 MPa and 250.5 MPa, while the A_0_ alloy’s yield strengths were 223 MPa and 217.5 MPa. This improvement in high-temperature mechanical properties is due to two main factors. Firstly, TiC + TiB2 particles refine the microstructure and interact with the strengthening T_1_ precipitation phase. They act as heterogeneous nucleation sites, promoting T_1_ phase precipitation and preventing its coarsening during high-temperature ageing. Secondly, TiC + TiB_2_ particles interact with dislocations, forming Orowan dislocation rings, further restraining dislocation movement.
3. Integrating TiC + TiB_2_ particles significantly improves aluminium–lithium alloys. It refines grain size and boosts toughness, strength, and dislocation density, strengthening alloys via dislocations. TiC + TiB_2_ inhibits T_1_ phase coarsening, keeping fine needle-like T_1_ phases (primary reinforcing phases) that enhance the properties. As a result, alloys with TiC + TiB_2_ have much higher yield and tensile strengths and are stable at high temps. Also, TiC + TiB_2_ increases alloy toughness, creating materials with excellent strength/toughness and expanding application potential.

## Figures and Tables

**Table 1:** Chemical compositions (wt%) of the three alloys.

Element	Li	Cu	Mg	Ag	Zr	Mn	Zn	TiC/TiB_2_	Al
2055	1.2	3.7	0.4	0.4	0.11	0.3	0.5	0	Bal.
0.5	1.2	3.7	0.4	0.4	0.11	0.3	0.5	0.5	Bal.
1.0	1.2	3.7	0.4	0.4	0.11	0.3	0.5	1.0	Bal.

**Table 2:** Physical properties of main ceramic phases.

Reinforcing Patricle	Density	Melting Point (°C)	The Expansion Coefficient	Vickers Hardness (HV)	Elastic Modulus (MPa)
TiC	4.25	3140	7.4	28–35	510
TiB_2_	4.5	2980	6.8	33	480–563

**Table 3:** The yield and tensile strengths of the four samples.

	Yield Elongation (%)	Yield Strength (MPa)	Tensile Elongation (%)	Tensile Strength (MPa)
AA2055 + 24 h	0.8025	222.22 ± 2	6.52	308.13 ± 2
AA2055 + 48 h	0.8012	217.56 ± 2	6.25	296.13 ± 2
AA2055 + 1.0% TiC/TiB_2_ + 24 h	0.8032	256.54 ± 2	5.99	336.86 ± 2
AA2055 + 1.0% TiC/TiB_2_ + 48 h	0.8025	250.56 ± 2	5.99	326.11 ± 2

## Data Availability

The original contributions presented in the study are included in the article, further inquiries can be directed to the corresponding author.
